# Early Tumor Shrinkage and Clinical Outcomes for Esophageal Squamous Cell Carcinoma Patients Treated with Immune Checkpoint Inhibitors: Real-World Observational Study

**DOI:** 10.1245/s10434-026-19849-x

**Published:** 2026-05-27

**Authors:** Keisuke Kosumi, Kazuto Harada, Takuya Shimogawa, Hiroki Tsubakihara, Yoshihiro Hara, Chihiro Matsumoto, Kohei Yamashita, Mayuko Ohuchi, Kojiro Eto, Katsuhiro Ogawa, Satoshi Ida, Yukiharu Hiyoshi, Yohei Nagai, Yoshifumi Baba, Yuji Miyamoto, Naoya Yoshida, Masaaki Iwatsuki

**Affiliations:** 1 Department of Gastroenterological Surgery, Graduate School of Medical Sciences, Kumamoto University, Kumamoto, Japan; 2 Department of Gastrointestinal Surgery, Graduate School of Medicine, The University of Tokyo, Tokyo, Japan

**Keywords:** Early tumor shrinkage, Esophageal neoplasms, Immune checkpoint, Immunity, Predictive marker

## Abstract

**Background:**

Immune checkpoint inhibitors (ICIs) have become a viable treatment method for unresectable or recurrent esophageal squamous cell carcinoma (ESCC). However, the relationships between early tumor shrinkage (ETS) and clinical outcomes for ESCC patients receiving ICIs remain unknown. This observational study investigated the associations between ETS and clinical outcomes for patients with unresectable or recurrent ESCC who received ICIs.

**Methods:**

Early tumor shrinkage was defined as the percentage decreases at the first evaluation, and the ETS cutoff value was 20%. Using an unbiased database of 128 unresectable or recurrent ESCC patients who received ICI plus chemotherapy or ICI plus ICI, this study evaluated the relationships between ETS and clinical outcomes by multivariable Cox proportional hazards regression models to adjust for potential confounders, including the clinical and pathologic features.

**Results:**

Among the patients, 52% had an ETS of 20% or more, and 48% had an ETS smaller than 20%. The ETS was not significantly associated with any of the clinical or pathologic features examined (all *P* > 0.09). However, ETS was significantly associated with longer progression-free survival (*P* < 0.01). Compared with the ETS-absent group, the multivariable-adjusted hazard ratio (HR) for the ETS-present group was 0.23 (95% confidence interval [Cl], 0.14–0.39; *P* < 0.01). Similarly, the same trend was observed for overall survival (multivariable-adjusted HR, 0.15; 95% CI, 0.07–0.31; *P* < 0.01). These prognostic associations of ETS were similarly observed in the ICI+chemotherapy and ICI+ICI groups (all *P* < 0.01).

**Conclusions:**

Early tumor shrinkage was associated with favorable clinical outcomes for ICI-treated patients with unresectable or recurrent ESCC, providing a platform for further investigations of predictive markers and treatment strategies.

**Supplementary Information:**

The online version contains supplementary material available at 10.1245/s10434-026-19849-x.

Growing evidence has highlighted the pivotal roles of immune cells in the tumor microenvironment in regulating carcinogenesis and tumor progression. Furthermore, substantial data have indicated that a stronger immune response to the tumor is associated with improved clinical outcomes for patients with various cancer types, including esophageal squamous cell carcinoma (ESCC).^1^ Progressive insights into tumor immunology and the immunosuppressive microenvironment favoring tumor development have facilitated the advent of immunotherapy targeting immune checkpoint molecules, including programmed death 1 (PDCD1, PD-1), programmed death-ligand 1 (PDCD1 ligand 1, CD274, PD-L1), and cytotoxic T-lymphocyte-associated protein 4 (CTLA4) in ESCC patients.^2,3^

The ATTRACTION-3 trial evaluated the efficacy and safety of nivolumab versus chemotherapy as a second-line treatment for advanced ESCC. The results demonstrated that nivolumab provided a clinically significant long-term improvement in overall survival (OS) compared with chemotherapy.^4–6^ Furthermore, results from the KEYNOTE-590 trial demonstrated the effectiveness of pembrolizumab combined with chemotherapy as a first-line treatment for patients with untreated locally advanced, unresectable, or metastatic ESCC or esophageal adenocarcinoma.^7–9^

From the results of CheckMate 648, nivolumab plus ipilimumab combination therapy has been administered as a first-line treatment in Japan since 2022 for patients with unresectable progressive or recurrent ESCC.^10–12^ The Japanese Esophageal Society has provided strong evidence supporting the recommendation of pembrolizumab combined with chemotherapy and nivolumab plus ipilimumab, as well as cisplatin plus 5-fluorouracil (5-FU), as first-line treatments for unresectable, advanced, or recurrent esophageal cancer.^13^

Data from various clinical trials have verified the safety and efficacy of immune checkpoint inhibitors (ICIs), indicating that immune checkpoint blockade is a promising strategy for the first- and second-line treatment of advanced esophageal cancer. However, the proportion of patients achieving sustained responses remains limited,^7–11^ highlighting the critical need to identify predictive markers of long-term efficacy.

Early tumor shrinkage (ETS) and depth of response (DpR) may serve as novel, easily assessable radiologic parameters for evaluating tumor response dynamics, with both ETS and DpR demonstrating strong correlations with survival outcomes in metastatic colorectal cancers.^14^ For esophageal cancer patients with metastatic disease undergoing chemotherapy, ETS and DpR are potentially associated with clinical outcomes.^15^ Emerging evidence suggests that ETS and DpR are crucial prognostic factors for patients who have metastatic cancers treated with ICIs and might be useful for designing strategies to intensify treatment.^16–18^ Whereas assessment of DpR is more likely to be delayed because the time to maximum shrinkage is longer and unpredictable, ETS can be examined at a specified early time point after treatment initiation. Therefore, ETS is more useful than DpR as an early on-treatment predictive marker.^15^ However, whether ETS can serve as a predictor of clinical outcomes or not for patients receiving immunotherapy-based treatments for ESCC remains unclear.

The purpose of the current study was to investigate the associations between ETS and clinical outcomes for patients with unresectable or recurrent ESCC who received ICIs. From the currently available evidence, we hypothesized that ETS is associated with improved clinical outcomes for patients with untreated unresectable or recurrent ESCC who received ICIs. To test this hypothesis, we used an unbiased clinical and pathologic epidemiology database of ESCC patients to examine prognostic markers for individuals who had recurrent or unresectable ESCC treated with ICIs.

A growing body of evidence has shown complex links among intestinal tumorigenesis, immunity, the gut microbiome, and patient characteristics. Esophageal cancer comprises heterogeneous processes with differing sets of genetic and epigenetic alterations, which are influenced by diet, environmental and microbial exposure, and host immunity. The molecular heterogeneity of the tumor cells, immune cell infiltrates, and bacteria in the tumor microenvironment poses a significant challenge to the clinical efficacy of treatment methods. Furthermore, the interactions among the tumor, host immune system, and patient characteristics in human cancers are complex, thereby hindering the development of *in vitro* and *in vivo* models that faithfully reproduce these interrelationships. Consequently, analyses of the tumor characteristics, immune cells, and clinicopathologic features of human cancer tissues are critically important.

## Materials and Methods

### Study Population

This retrospective cohort study investigated patients with unresectable or recurrent ESCC who received ICIs at Kumamoto University Hospital. Our unbiased clinical and pathologic epidemiology database includes all consecutive patients treated with ICIs at our institution. Eligible patients were 18 years of age or older with previously untreated, histologically or cytologically confirmed, unresectable advanced, recurrent, or metastatic ESCC.

This observational study enrolled 128 patients with recurrent or unresectable ESCC cancer who were treated with pembrolizumab+CDDP+5-FU, nivolumab+CDDP+5-FU, or nivolumab+ipilimumab at Kumamoto University Hospital between December 2021 and October 2024. In our hospital, pembrolizumab+CDDP+5-FU was authorized for the treatment of esophageal cancer patients in December 2021, whereas nivolumab+CDDP+5-FU and nivolumab+ipilimumab were authorized in May 2022. Patients were observed at 1- to 3-month intervals until death or 31 December 2024, whichever came first.

This study was conducted as a retrospective observational study. Written informed consent was obtained from each subject whenever feasible. For cases in which obtaining written consent was not possible, including deceased patients, an opt-out consent process was applied in accordance with the ethical guidelines of our institution, whereby information regarding the study was publicly disclosed and patients or their representatives were given the opportunity to decline participation.

The study procedures were reviewed and approved by the Institutional Review Board of Kumamoto University (IRB no. 1909). The term “prognostic marker’’ is used throughout this article according to the REMARK Guidelines.^19^ The study was performed in accordance with the Declaration of Helsinki.

### Treatment Procedures

Patients received pembrolizumab (200 mg every 3 weeks) plus chemotherapy (3-week cycle of fluorouracil 800 mg/m^2^ on days 1 to 5 and cisplatin 80 mg/m^2^ on day 1), nivolumab (480 mg every 4 weeks) plus chemotherapy (4-week cycle of fluorouracil 800 mg/m^2^ on days 1 to 5 and cisplatin 80 mg/m^2^ on day 1), or nivolumab (360 mg every 3 weeks) plus ipilimumab (1 mg/kg every 6 weeks).^7–11^ Treatment was continued until disease progression, toxicity requiring treatment discontinuation, or withdrawal of consent. Patients could continue treatment beyond the initial disease progression based on the investigators’ judgment.^6,20^

Patients were followed up with outpatient visits after each treatment cycle. Tumor response was assessed using computed tomography (CT) according to the Response Evaluation Criteria in Solid Tumors (RECIST) version 1.1 at baseline and every 8 to 10 weeks thereafter, until the initiation of poststudy treatment or disease progression. Formal tumor assessments were performed every two treatment cycles. The initial treatment response was evaluated using positron emission tomography (PET)-contrast-enhanced CT, whereas subsequent evaluations were conducted using contrast-enhanced CT scans with a slice thickness of 5 mm. Tumor measurements were performed by physicians who were not involved in the patients’ direct clinical care and were blinded to the clinical outcomes. Early tumor shrinkage was defined as the percentage decrease in the sum of the target lesion sizes at the first evaluation compared with that at baseline, and the ETS cutoff value was 20%.^8,21^ Progression-free survival (PFS) was defined as the interval from the initiation of ICI treatment to evidence of progressive disease (PD) or death from any cause, whichever occurred first. Overall survival (OS) was defined as the interval from the initiation of ICI treatment to death from any cause.

### Statistical Analysis

All statistical analyses were performed using the JMP program (version 16; SAS Institute, Cary, NC, USA). All *P* values were two-sided, with a two-sided alpha level of 0.05 used for all testing. The primary hypothesis testing was for the associations of ETS with PFS and OS as an outcome variable. The Kaplan-Meier method was used to describe the distribution of PFS and OS, and the log-rank test was used to compare survival probabilities across patient characteristics. A Cox proportional hazards model was used to compute the hazard ratios (HRs) and 95% confidence intervals (CIs). Multivariable Cox proportional hazards regression models were used to identify the independent risk factors for PFS and OS. The multivariable models included variables that showed a univariable association (*P* < 0.05) with PFS and OS. All other analyses represented secondary analyses.

To compare characteristics across the strata of ICIs, the chi-square test was used for categorical variables. Analysis of variance, assuming equal variances, was used for continuous variables. Each of the cross-sectional analyses were secondary.

## Results

### Clinical and Pathologic Characteristics of ESCC Cases Treated with ICIs

Table 1 summarizes the clinical and pathologic features of the patients who received ICI+chemotherapy or ICI+ICI for ESCC. In an unbiased independent database comprising 128 ESCC patients, the mean age was 67 years, and 81% of the patients were male. Histologically, all cancer was squamous cell carcinoma (SCC) (128 cases, 100%). The most frequent indication for ICI was recurrence (65 cases, 51%), followed by unresectable cancer (63 cases, 49%). Blood testing at the initiation of ICI treatment showed slight elevations of C-reactive protein and SCC antigen levels.

**Table 1:** Clinical and pathologic characteristics of esophageal squamous cell carcinoma cases with ICIs

Characteristic^a^	All cases (*n* = 128) *n* (%)	ICI+chemotherapy (*n* = 81) *n* (%)	ICI+ICI (*n* = 47) *n* (%)
Mean age (years)	67 ± 8.7	66 ± 9.0	68 ± 8.1
*Sex*			
Male	104 (81)	64 (79)	40 (85)
Female	24 (19)	17 (21)	7 (15)
Mean BMI (kg/m^2^)	20 ± 3.1	20 ± 2.9	20 ± 3.4
*ECOG PS*			
0	116 (91)	79 (98)	37 (79)
1	8 (6.3)	2 (2.5)	6 (13)
2	4 (3.1)	–	4 (8.5)
*Tumor location*			
Ce	19 (15)	8 (9.9)	11 (23)
Te	109 (85)	73 (90)	36 (77)
*Histologic type*			
Squamous cell carcinoma	128 (100)	81 (100)	47 (100)
*PD-L1 TPS*			
0≤TPS<1	23 (38)	10 (40)	12 (36)
1≤TPS	38 (62)	15 (60)	23 (64)
*Indication*			
Unresectable	63 (49)	40 (49)	23 (49)
Recurrence	65 (51)	41 (51)	24 (51)
*Treatment line*			
1	120 (94%)	79 (98%)	41 (87%)
2≤	8 (6.3%)	2 (2.5%)	6 (13%)
Mean albumin (g/dL)	3.6 ± 0.5	3.7 ± 0.5	3.5 ± 0.6
Mean creatinine (mg/dL)	0.8 ± 0.2	0.8 ± 0.2	0.9 ± 0.3
Mean C-reactive protein (mg/dL)	1.3 ± 2.0	1.1 ± 1.6	1.8 ± 2.4
Mean neutrophil-to-lymphocyte ratio	4.3 ± 3.2	3.7 ± 2.4	5.3 ± 4.1
Mean SCC antigen level (ng/mL)	3.4 ± 6.2	3.8 ± 7.5	2.7 ± 3.0

ICI, immune checkpoint inhibitor; BMI, body mass index; ECOG PS, Eastern Cooperative Oncology Group performance status; PD-L1, programmed death-ligand 1; TPS, tumor proportion score; SCC, squamous cell carcinoma

^a^Percentage indicates the proportion of patients with a specific clinical, pathologic, or molecular characteristic among all the patients

### Clinical Outcomes of ESCC Patients Treated with ICIs

Table 2 summarizes the clinical outcomes for the patients who received ICI+chemotherapy or ICI+ICI for ESCC. Among these cases, at the first evaluation by CT, three patients (2.8%) achieved a complete response and 44 patients (42%) achieved a partial response. The primary response rates were 45% for all the patients, 49% for the patients treated with ICI+chemotherapy, and 37% for the patients treated with ICI+ICI. The best overall response rates were 53% for all the patients, 60% for the patients treated with ICI+chemotherapy, and 42% for the patients treated with ICI+ICI. The best disease control rates were 79% for all the patients, 83% for the patients treated with ICI+chemotherapy, and 72% for the patients treated with ICI+ICI.

**Table 2:** Clinical outcomes of esophageal squamous cell carcinoma cases with ICIs

Characteristic^a^	All cases (*n* = 128) *n* (%)	ICI+chemotherapy (*n* = 81) *n* (%)	ICI+ICI (*n* = 47) *n* (%)
*Initial efficacy assessment*			
CR	3 (2.9)	1 (1.5)	2 (5.7)
PR	44 (42)	33 (48)	11 (31)
SD	35 (34)	23 (33)	12 (34)
PD	22 (21)	12 (17)	10 (29)
*Best overall response*			
CR	19 (18)	13 (19)	6 (17)
PR	37 (35)	28 (41)	9 (25)
SD	27 (26)	16 (23)	11 (31)
PD	22 (21)	12 (17)	10 (28)
Overall response rate (CR+PR) (%)	53	59	42
Disease control rate (CR+PR+SD) (%)	79	83	72

ICI, immune checkpoint inhibitor; CR, complete response; PR, partial response; SD, stable disease; PD, progressive disease; PFS, progression-free survival

^a^Percentage indicates the proportion of patients with a specific clinical, pathologic, or molecular characteristic among all the patients

**Fig. 1:**
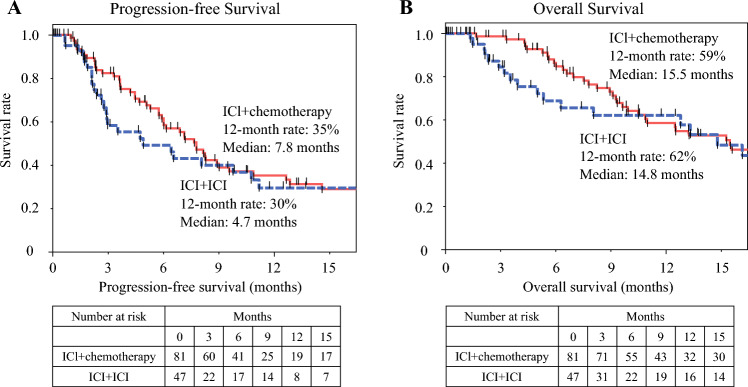
Kaplan–Meier estimates of progression-free survival and overall survival of ESCC patients with ICI regimens. **A** Progression-free survival of patients with ICI+chemotherapy and ICI+ICI. **B** Overall survival of patients with ICI+chemotherapy and ICI+ICI. ESCC, esophageal squamous cell carcinoma; ICI, immune checkpoint inhibitor

With a median follow-up period of 11.3 months for the patients treated with ICI+chemotherapy, the median PFS and OS values were 7.8 months and 15.5 months, respectively (Fig. 1A and B). With a median follow-up period of 8.4 months for the patients treated with ICI+ICI, the median PFS and OS values were 4.7 months and 14.8 months, respectively (Fig. 1A and B).

### The Association of ETS with Clinical and Pathologic Characteristics, and Clinical Outcomes for ESCC Patients Treated With ICIs

Table 3 summarizes the clinical and pathologic features of the patients stratified according to their ETS status. Among 102 cases, 52% had an ETS of 20% or more, and 48% had an ETS smaller than 20%. The findings showed that ETS was not significantly associated with any of the features examined (all *P* > 0.09).

**Table 3:** Clinical and pathologic characteristics of esophageal squamous cell carcinoma cases with ICIs

Characteristic^a^	All cases (*n* = 102) *n* (%)	Non-ETS (*n* = 53) *n* (%)	ETS (*n* = 49) *n* (%)	*P* value
Mean age (years)	67 ± 8.2	68 ± 7.1	66 ± 9.2	0.30
Sex				0.48
Male	84 (82)	45 (85)	39 (80)	
Female	18 (18)	8 (15)	10 (20)	
Mean BMI (kg/m^2^)	20 ± 3.0	20 ± 3.3	20 ± 2.8	0.38
ECOG PS				0.097
0	96 (94)	48 (91)	48 (98)	
1	3 (2.9)	3 (5.7)	–	
2	3 (2.9)	2 (3.4)	1 (2.0)	
Tumor location				0.64
Ce	12 (12)	7 (13)	5 (10)	
Te	90 (88)	46 (87)	44 (90)	
Histologic type				-
Squamous cell carcinoma	102 (100)	53 (100)	49 (100)	
PD-L1 TPS				0.78
0≤TPS<1	14 (30)	7 (28)	7 (32)	
1≤TPS	33 (70)	18 (72)	15 (68)	
Indication				0.24
Unresectable	52 (51)	30 (57)	22 (45)	
Recurrence	50 (49)	23 (43)	27 (55)	
Treatment line				0.18
1	97 (95)	49 (92)	48 (98)	
≥2	5 (4.9)	4 (7.6)	1 (2.0)	
Mean albumin (g/dL)	3.6 ± 0.5	3.6 ± 0.6	3.7 ± 0.5	0.33
Mean creatinine (mg/dL)	0.8 ± 0.2	0.9 ± 0.2	0.8 ± 0.3	0.46
Mean C-reactive protein (mg/dL)	1.3 ± 2.0	1.6 ± 2.3	1.0 ± 1.6	0.11
Mean SCC antigen level (ng/mL)	2.9 ± 3.7	2.8 ± 3.9	3.0 ± 3.4	0.82
Mean neutrophil-to-lymphocyte ratio	4.3 ± 3.2	4.5 ± 2.9	3.8 ± 3.2	0.26
Mean lymphocyte-to-monocyte ratio	3.9 ± 6.8	4.4 ± 10.4	3.8 ± 1.7	0.70
Mean platelet-to-lymphocyte ratio	270 ± 218	271 ± 159	269 ± 286	0.97
Mean systemic immune-inflammation index	114 ± 102	116 ± 91	106 ± 109	0.63
Mean C-reactive protein to albumin ratio	0.43 ± 0.70	0.54 ± 0.85	0.30 ± 0.53	0.10
Mean prognostic nutritional index	42 ± 6.8	42 ± 6.8	44 ± 6.5	0.14

ETS, early tumor shrinkage; BMI, body mass index; ECOG PS, Eastern Cooperative Oncology Group performance status; PD-L1, programmed death-ligand 1; TPS, tumor proportion score; SCC, squamous cell carcinoma

^a^Percentage indicates the proportion of patients with a specific clinical, pathological, or molecular characteristic among all patients

**Fig. 2:**
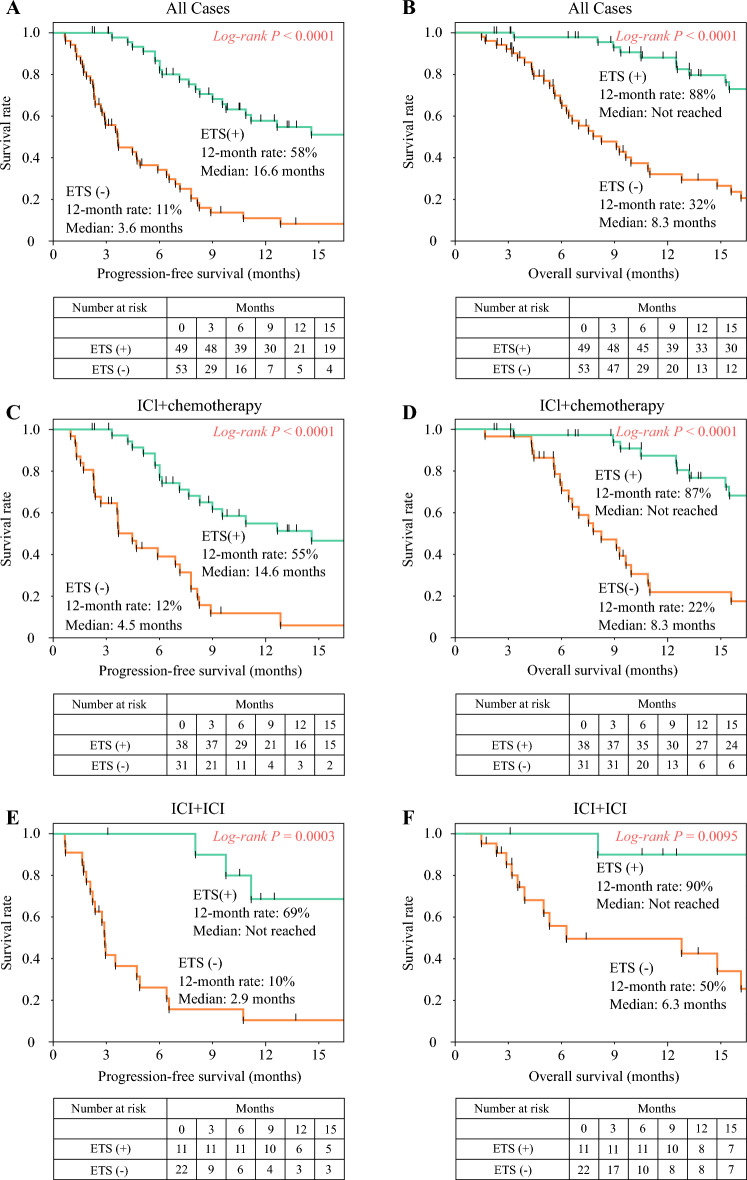
ETS and clinical outcomes for ESCC patients with ICI regimens. **A** PFS according to ETS for patients with unresectable or recurrent ESCC who received ICI regimens. **B** OS according to ETS for patients with unresectable or recurrent ESCC who received ICI regimens. **C,E** PFS according to ETS for patients with unresectable or recurrent ESCC who received **C** ICI+chemotherapy and **E** ICI+ICI. **D,F** OS according to ETS for patients with unresectable or recurrent ESCC who received **D** ICI+chemotherapy and **F** ICI+ICI. ETS, early tumor shrinkage; ESCC, esophageal squamous cell carcinoma; ICI, immune checkpoint inhibitor; PFS, progression-free survival; OS, overall survival

To test our primary hypothesis, we assessed the association of ETS with the clinical outcomes for the ESCC patients who received ICIs. The univariable Cox regression models suggested that good Eastern Cooperative Oncology Group performance status (ECOG PS), thoracic ESCC, recurrence, and ETS were significantly associated with longer PFS (all *P* < 0.05; Table 4). The multivariable Cox regression model showed that ETS was significantly associated with longer PFS (*P* < 0.0001). In the univariable model, for ETS in the PFS outcome category, the HR was 0.23 (95% CI, 0.14–0.39; *P* < 0.0001; Table 4). In the multivariable model, which controlled for potential confounders displaying an association in the univariable analysis (*P* < 0.05), the multivariable HR was 0.22 (95% CI, 0.13–0.37; *P* < 0.0001; Table 4). Thoracic ESCC and recurrence also may be associated with longer PFS (*P* < 0.02). In the Kaplan-Meier survival analyses, ETS was associated with longer PFS in all cases (*P* < 0.0001; Fig. 2A).

**Table 4:** ETS and PFS among esophageal squamous cell carcinoma cases with ICI regimens

	PFS				OS			
	Univariable HR		Multivariable HR		Univariable HR		Multivariable HR	
	(95% CI)	*P* Value	(95% CI)^a^	*P* Value	(95% CI)	*P* Value	(95% CI)^a^	*P* Value
Age (for 10-year increment)	0.82 (0.63–1.07)	0.14			0.98 (0.71–1.37)	0.92		
*Sex*								
Female	1 (referent)	0.52			1 (referent)	0.80		
Male	0.84 (0.49–1.44)				1.09 (0.56–2.13)			
BMI (for 1-kg/m^2^ increment)	0.93 (0.87–1.00)	0.055			0.88 (0.80–0.96)	0.0039	0.92 (0.83–1.02)	0.12
*ECOG PS*								
0	1 (referent)	0.045	1 (referent)	0.24	1 (referent)	<0.0001	1 (referent)	0.029
1–2	2.41 (1.02–5.69)		1.88 (0.66–5.39)		7.81 (2.86–21.3)		4.56 (1.17–17.7)	
*Tumor location*								
Ce	1 (referent)	0.0024	1 (referent)	0.018	1 (referent)	0.30		
Te	0.37 (0.20–0.70)		0.39 (0.18–0.85)		0.64 (0.27–1.50)			
*PD-L1 TPS*								
0≤TPS<1	1 (referent)	0.26			1 (referent)	0.99		
1≤TPS	1.58 (0.72–3.50)				1.01 (0.39–2.61)			
*Indication*								
Unresectable	1 (referent)	0.0056	1 (referent)	0.014	1 (referent)	0.024	1 (referent)	0.29
Recurrence	0.52 (0.32–0.82)		0.53 (0.32–0.88)		0.52 (0.30–0.92)		0.72 (0.39–1.33)	
*Treatment line*								
1	1 (referent)	0.87			1 (referent)	0.56		
≤2	0.92 (0.34–2.52)				1.36 (0.49–3.78)			
Albumin (for 1-g/dL increment)	0.93 (0.60–1.48)	0.77			0.49 (0.29–0.84)	0.0082	0.73 (0.37–1.46)	0.36
Creatinine (for 1-mg/dL increment)	0.68 (0.26–1.71)	0.42			0.60 (0.18–1.88)	0.39		
C-reactive protein (for 1-mg/dL increment)	1.11 (0.97–1.24)	0.099			1.12 (0.94–1.28)	0.16		
SCC antigen level (for 1-ng/mL increment)	1.01 (0.97–1.04)	0.44			1.04 (1.00–1.06)	0.018	1.07 (0.99–1.14)	0.063
*Early tumor shrinkage*								
Absent	1 (referent)	<0.0001	1 (referent)	<0.0001	1 (referent)	<0.0001	1 (referent)	<0.0001
Present	0.23 (0.14–0.39)		0.22 (0.13–0.37)		0.21 (0.11–0.40)		0.15 (0.07–0.31)	

ETS, early tumor shrinkage; PFS, progression-free survival; ICI, immune checkpoint inhibitor; OS, overall survival; HR, hazard ratio; CI, confidence interval; BMI, body mass index; ECOG PS, Eastern Cooperative Oncology Group performance status; PD-L1, programmed death-ligand 1; TPS, tumor proportion score; SCC, squamous cell carcinoma

^a^Multivariable Cox proportional hazards regression models were used to identify independent risk factors for PFS. The multivariable models included variables showing a univariable association (*P* < 0.05) with PFS

The univariable Cox regression models suggested that a high body mass index (BMI), good ECOG PS, recurrence, high albumin level, low SCC antigen level, and ETS were significantly associated with longer OS (all *P* < 0.03; Table 4). The multivariable Cox regression model showed that ETS was significantly associated with longer OS (*P* < 0.0001). In the univariable model, for ETS in the OS outcome category, the HR was 0.21 (95% CI, 0.11–0.40; *P* < 0.0001; Table 4). In the multivariable model, which controlled for potential confounders displaying an association in the univariable analysis (*P* < 0.05), the multivariable HR was 0.15 (95% CI, 0.07–0.31; *P* < 0.0001; Table 4). Good ECOG PS also may be associated with longer OS (*P* = 0.029). In the Kaplan-Meier survival analyses, ETS was associated with longer OS in all cases (*P* < 0.0001; Fig. 2B).

As exploratory analyses, we also performed Kaplan-Meier survival analyses according to ICI regimen. As a result, ETS was still significantly associated with longer PFS in the ICI+chemotherapy and ICI+ICI groups (all *P* < 0.01; Fig. 2C and E). Similarly, the same trend was observed for OS, with ETS significantly associated with longer OS in both the ICI+chemotherapy and ICI+ICI groups (all *P* < 0.01; Fig. 2D and F).

## Discussion

Immune cells within the tumor microenvironment have been identified as a prognostic factor for esophageal cancer.^1^ The advent of immunotherapies has revolutionized treatment strategies for esophageal cancer, significantly improving patient survival rates.^22,23^ Although ETS has been associated with favorable outcomes for cancer patients treated with ICIs, its prognostic role in esophageal cancer remains unclear. To address this, we hypothesized that ETS may be associated with clinical outcomes for patients with unresectable or recurrent esophageal cancer undergoing ICI therapy. Using a molecular pathologic epidemiology database of 130 esophageal cancer cases, we observed that ETS was linked to improved clinical outcomes, suggesting that ETS is a potential prognostic marker for this disease (Table 5).

**Table 5:** Overview studies on early tumor shrinkage in various cancer patients treated with ICI regimens

Tumor type	Sample size	Treatment	ETS cutoff (%)	Evaluation time	Outcome	References
ESCC	128	ICI+chemotherapy,ICI+ICI	20	6–10 Weeks	PFS, HR = 0.22 OS, HR = 0.15	Current study
Esophageal cancer	19	Nivolumab+ipilimumab	20	2 Months	PFS, HR = 0.15	Natsuki S (2025)^24^
HCC	157	TACE+lenvatinib+toripalimab	10	6 Weeks	PFS, HR = 0.39 OS, HR = 0.58	Zhang X (2025)^26^
ESCC	53	Pembrolizumab+chemotherapy	20	2 Cycles	PFS, HR = 0.15	Sugase T (2024)^21^
Esophageal cancer	141	Pembrolizumab+chemotherapy (KEYNOTE-590)	20	8–10 Weeks	PFS, HR = 0.24 OS, HR = 0.233	Kato K (2024)^8^
HER2-negative advanced GC	136	Nivolumab+chemotherapy	20	6–10 Weeks	PFS, HR = 0.34 OS, HR = 0.47	Shimozaki K (2024)^25^
MSI-high mCRC	169	Anti-PD-1 monotherapy, anti-PD-1+anti-CTLA-4 agents	1	8–9 Weeks	PFS, HR = 0.26 OS, HR = 0.35	Fucà G (2021)^16^
NSCLC	1942	Atezolizumab	10	6 Weeks	PFS, HR = 0.31 OS, HR=0.32	Hopkins AM (2020)^17^
Melanoma	452	Pembrolizumab (KEYNOTE-001, –002, –006)	10	12–24 Weeks	OS^a^	Wang M (2019)^18^
NSCLC	83	Nivolumab	10	8–12 Weeks	PFS, 16.6 months (8.5 months–NR) vs 5.1 months (3.9–6.8 months) OS, NR (NR–NR) vs NR (15.3 months–NR)	Kawachi H (2019)^27^

ICI, immune checkpoint inhibitor; ETS, early tumor shrinkage; ESCC, esophageal squamous cell carcinoma; PFS, progression-free survival; HR, hazard ratio; OS, overall survival; HCC, hepatocellular carcinoma; TACE, transarterial chemoembolization; HER2, human epidermal growth factor receptor 2; GC, gastric cancer; MSI, microsatellite instability; CRC, colorectal cancer; mCRC, metastatic colorectal cancer; PD-1, programmed death-1; CTLA-4, cytotoxic T-lymphocyte-associated protein 4; NSCLC, non-small cell lung cancer; NR, not reached

^a^This study does not present data on hazard ratios or median survival differences

Accumulating evidence has shown a relationship between ETS and cancer patient prognosis. Early tumor shrinkage shows a strong association with survival outcomes for metastatic colorectal cancer patients (mCRC) receiving first-line treatment, with one large retrospective cohort study also providing new evidence on the prognostic impact of ETS in patients with MSI­H/dMMR mCRC receiving ICIs.^16^ In esophageal cancer, one cohort study demonstrated a significant association between ETS and long-term survival for patients who had metastatic or recurrent esophageal cancer treated with two-weekly docetaxel combined with cisplatin plus fluorouracil.^15^

Our current human institution-based study showed an independent relationship between ETS and favorable clinical outcomes for ESCC patients who received ICIs. Furthermore, our findings on the correlation between ETS and survival outcomes align with previously reported data on patients with several tumor types receiving ICIs.^8,16–18,21,24–27^ Early tumor shrinkage may be a potentially novel, easy-evaluable, on-treatment radiologic parameter for assessing the dynamics of tumor response in patients with unresectable or metastatic ESCC treated with ICIs. Patients who fail to achieve ETS may represent a high-risk subgroup that could benefit from closer monitoring or early consideration of alternative therapeutic strategies. Further large-scale prospective studies are required to clarify the complex relationship between ETS and clinical outcomes and to determine whether ETS-guided treatment modification can improve patient management.

A growing number of studies have investigated predictive markers in various cancers after ICI therapy. In one retrospective study, potential associations were observed between esophageal cancer patients with a lower neutrophil-to lymphocyte ratio (NLR) and better performance status before treatment with the benefits of ICIs.^28^ Another study investigated the predictive value of certain inflammatory markers, including the NLR, platelet-to-lymphocyte ratio (PLR), lymphocyte-to-monocyte ratio (LMR), systemic immune-inflammation index (SII), and prognostic nutritional index (PNI), for locally advanced or recurrent/metastatic cervical cancer patients treated with ICIs. This study highlighted the potential use of SII as a predictor of PFS for these patients.^29^ Carroll et al.^30^ demonstrated that a 4-week course of ICIs is sufficient to induce tumor shrinkage in esophageal adenocarcinoma patients exhibiting upregulation of the ‘‘INCITE’’ gene signature. The integration of single-cell RNA-sequencing and bulk RNA-sequencing through deconvolution identified the tumor-associated monocyte content and tumor mutational burden as independent, yet complementary, predictive biomarkers for immunochemotherapy.

As a next step, further research is warranted to elucidate the underlying mechanisms linking ETS to favorable clinical outcomes and to identify robust predictive biomarkers for ETS. Although the exact mechanisms remain to be fully elucidated, several hypotheses have been proposed. First, ETS during ICI therapy may reflect early and effective activation of antitumor immunity because ETS has been consistently associated with improved survival outcomes across multiple tumor types treated with ICIs. Second, ETS has been reported to correlate with systemic inflammatory status, such as lower neutrophil-to-lymphocyte ratios. In the current study, although not statistically significant, there was a trend suggesting that ETS may be associated with better ECOG PS and a lower C-reactive protein-to-albumin ratio, suggesting that a favorable immune microenvironment may facilitate early tumor regression. However, in the current study, multivariable logistic regression analysis did not demonstrate a significant association between ETS and either ECOG PS or the C-reactive protein-to-albumin ratio (Table S1). Collectively, ETS may serve as an early radiologic surrogate of effective immune activation and tumor microenvironment modulation during ICI therapy. Emerging evidence has indicated that CT radiomic studies in esophageal cancer patients can improve the identification of early-stage disease and prediction of treatment response.^31,32^ Radiogenomics provides clinically useful prognostic predictions by linking molecular characteristics, such as gene mutations and gene expression patterns in malignant tumors, with medical images, leading to more opportunities for disease management.^33^

We acknowledge several limitations of our study. First, the current study was a retrospective, single-center investigation with a small number of patients, and the follow-up duration was not adequate for assessing the long-term survival outcome. Because ICIs have been approved to treat esophageal cancer only a few years, our findings should be verified in a larger cohort in a multi-institutional joint study. Second, we did not consider molecular characteristics, such as the PD-L1 expression levels, in all these esophageal cancer tissues. Third, we did not clarify the exact mechanisms of the associations between ETS and favorable clinical outcomes. However, emerging evidence, including experimental studies and human studies in various cancers, could support our findings. Future studies are needed to confirm our findings and examine the association of ETS with long-term survival for esophageal cancer patients who received ICIs.

A further limitation of this study was that we did not identify any significant predictors of ETS despite evaluation of several clinical and laboratory parameters, including PD-L1 expression and systemic inflammatory and nutritional markers. Further studies with larger sample sizes are needed to clarify the factors associated with ETS.

The strength of our study was in its use of a molecular pathologic epidemiology^34,35^ database of ESCC cases in an independent cohort. This database integrates epidemiologic data and clinicopathologic features. Experimental systems cannot perfectly recapitulate the complex nature of the host, human tumors, and the immune system. Thus, large population-based analyses are crucial for understanding the relationship between ETS and patient survival. Our findings suggest that ETS could serve as a potential marker for predicting ICI efficacy in ESCC, but the exact mechanisms that underlie this during tumorigenesis and cancer progression are unknown. Future studies with larger, multi-institutional cohorts are required to validate our current findings and explore the potential mechanisms controlling the relationships between ETS and favorable clinical outcomes.

In conclusion, ETS is associated with improved clinical outcomes for patients who have unresectable or recurrent ESCC treated with ICIs. These findings pave the way for further research into the interplay between ETS and immune responses, offering potential strategies to optimize immunotherapy-based treatment methods for ESCC.

## Supplementary Information

Below is the link to the electronic supplementary material.

Supplementary file1 (DOCX 54 kb)

## References

[1] Zheng X, Song X, Shao Y, et al. Prognostic role of tumor-infiltrating lymphocytes in esophagus cancer: a meta-analysis. *Cell Physiol Biochem*. 2018;45:720–32. 29414812 10.1159/000487164

[2] Gao TT, Shan JH, Yang YX, et al. Comparative efficacy and safety of immunotherapy for patients with advanced or metastatic esophageal squamous cell carcinoma: a systematic review and network meta-analysis. *BMC Cancer*. 2022;22:992. 36115960 10.1186/s12885-022-10086-5 PMC9482734

[3] Kadono T, Yamamoto S, Hirose T, et al. Safety and short-term efficacy of preoperative FOLFOX therapy in patients with resectable esophageal squamous cell carcinoma who are ineligible for cisplatin. *Esophagus* 2023;20(1):109–15. 10.1007/s10388-022-00951-4 PMC9813081 36050607

[4] Kato K, Cho BC, Takahashi M, et al. Nivolumab versus chemotherapy in patients with advanced oesophageal squamous cell carcinoma refractory or intolerant to previous chemotherapy (ATTRACTION-3): a multicentre, randomised, open-label, phase 3 trial. *Lancet Oncol*. 2019;20:1506–17. 31582355 10.1016/S1470-2045(19)30626-6

[5] Takahashi M, Kato K, Okada M, et al. Nivolumab versus chemotherapy in Japanese patients with advanced esophageal squamous cell carcinoma: a subgroup analysis of a multicenter, randomized, open-label, phase 3 trial (ATTRACTION-3). *Esophagus*. 2021;18:90–9. 33170461 10.1007/s10388-020-00794-x PMC7794205

[6] Okada M, Kato K, Cho BC, et al. Three-year follow-up and response-survival relationship of nivolumab in previously treated patients with advanced esophageal squamous cell carcinoma (ATTRACTION-3). *Clin Cancer Res*. 2022;28:3277–86. 35294546 10.1158/1078-0432.CCR-21-0985 PMC9662935

[7] Sun JM, Shen L, Shah MA, et al. Pembrolizumab plus chemotherapy versus chemotherapy alone for first-line treatment of advanced oesophageal cancer (KEYNOTE-590): a randomised, placebo-controlled, phase 3 study. *Lancet*. 2021;398:759–71. 34454674 10.1016/S0140-6736(21)01234-4

[8] Kato K, Kojima T, Hara H, et al. First-line pembrolizumab plus chemotherapy for advanced/metastatic esophageal cancer: 1-year extended follow-up in the Japanese subgroup of the phase 3 KEYNOTE-590 study. *Esophagus*. 2024;21:306–18. 38607538 10.1007/s10388-024-01053-z PMC11199245

[9] Kojima T, Hara H, Tsuji A, et al. First-line pembrolizumab + chemotherapy in Japanese patients with advanced/metastatic esophageal cancer from KEYNOTE-590. *Esophagus*. 2022;19:683–92. 35668304 10.1007/s10388-022-00920-x PMC9436840

[10] Kato K, Doki Y, Ogata T, et al. First-line nivolumab plus ipilimumab or chemotherapy versus chemotherapy alone in advanced esophageal squamous cell carcinoma: a Japanese subgroup analysis of open-label, phase 3 trial (CheckMate 648/ONO-4538-50). *Esophagus*. 2023;20:291–301. 36401133 10.1007/s10388-022-00970-1 PMC10024660

[11] Kato K, Doki Y, Chau I, et al. Nivolumab plus chemotherapy or ipilimumab versus chemotherapy in patients with advanced esophageal squamous cell carcinoma (CheckMate 648): 29-month follow-up from a randomized, open-label, phase III trial. *Cancer Med*. 2024;13:e7235. 38716626 10.1002/cam4.7235 PMC11077338

[12] Doki Y, Ajani JA, Kato K, et al. Nivolumab combination therapy in advanced esophageal squamous cell carcinoma. *N Engl J Med*. 2022;386:449–62. 35108470 10.1056/NEJMoa2111380

[13] Kitagawa Y, Ishihara R, Ishikawa H, et al. Esophageal cancer practice guidelines 2022 edited by the Japan Esophageal Society: part 2. *Esophagus*. 2023;20:373–89. 36995449 10.1007/s10388-023-00994-1 PMC10235142

[14] Manca P, Corallo S, Randon G, et al. Impact of early tumor shrinkage and depth of response on the outcomes of panitumumab-based maintenance in patients with RAS wild-type metastatic colorectal cancer. *Eur J Cancer*. 2021;144:31–40. 33321462 10.1016/j.ejca.2020.11.017

[15] Ura T, Hironaka S, Tsubosa Y, et al. Early tumor shrinkage and depth of response in patients with metastatic esophageal cancer treated with 2-weekly docetaxel combined with cisplatin plus fluorouracil: an exploratory analysis of the JCOG0807. *Esophagus*. 2023;20:272–80. 36427158 10.1007/s10388-022-00968-9

[16] Fucà G, Corti F, Ambrosini M, et al. Prognostic impact of early tumor shrinkage and depth of response in patients with microsatellite instability-high metastatic colorectal cancer receiving immune checkpoint inhibitors. *J Immunother Cancer*. 2021;9(4):e002501. 10.1136/jitc-2021-002501 PMC8051394 33849927

[17] Hopkins AM, Kichenadasse G, Karapetis CS, Rowland A, Sorich MJ. Early tumor shrinkage identifies long-term disease control and survival in patients with lung cancer treated with atezolizumab. *J Immunother Cancer*. 2020;8(1):e000500. 10.1136/jitc-2019-000500 PMC7279663 32503948

[18] Wang M, Chen C, Jemielita T, et al. Are tumor size changes predictive of survival for checkpoint blockade based immunotherapy in metastatic melanoma? *J Immunother Cancer*. 2019;7:39. 30736858 10.1186/s40425-019-0513-4 PMC6368769

[19] McShane LM, Altman DG, Sauerbrei W, et al. Reporting recommendations for tumor marker prognostic studies (REMARK). *J Natl Cancer Inst*. 2005;97:1180–4. 16106022 10.1093/jnci/dji237

[20] Eisenhauer EA, Therasse P, Bogaerts J, et al. New response evaluation criteria in solid tumours: revised RECIST guideline (version 1.1). *Eur J Cancer*. 2009;45:228–47. 19097774 10.1016/j.ejca.2008.10.026

[21] Sugase T, Kanemura T, Takeoka T, et al. Clinical impact of early tumour shrinkage in metastatic or unresectable oesophageal cancer treated with pembrolizumab plus chemotherapy. *Oncology*. 2024;102:484–93. 38052183 10.1159/000535186 PMC11152033

[22] Ammannagari N, Atasoy A. Current status of immunotherapy and immune biomarkers in gastro-esophageal cancers. *J Gastrointest Oncol*. 2018;9:196–207. 29564185 10.21037/jgo.2017.06.12 PMC5848035

[23] Grierson P, Lim KH, Amin M. Immunotherapy in gastrointestinal cancers. *J Gastrointest Oncol*. 2017;8:474–84. 28736635 10.21037/jgo.2017.05.01 PMC5506270

[24] Natsuki S, Lee S, Kasashima H, et al. Utility of assessing early tumor shrinkage as an efficacy predictor in patients with non-surgically indicated or recurrent esophageal cancer treated with nivolumab plus ipilimumab. *Oncology*. 2025;103(3):167–78. 10.1159/000540851 39208777

[25] Shimozaki K, Fukuda K, Ooki A, et al. Safety and efficacy of first-line nivolumab plus chemotherapy for HER2-negative advanced gastric or gastroesophageal junction adenocarcinoma: real-world data analysis. *ESMO Gastrointest Oncol*. 2024;5:100072. 41647595 10.1016/j.esmogo.2024.100072 PMC12836541

[26] Zhang X, Shen Z, Qu S, Pan H, Chen Y, Wu D. Effect of early tumor shrinkage and depth of response on the clinical outcomes of patients with unresectable hepatocellular carcinoma treated with transcatheter arterial chemoembolization and lenvatinib plus PD-1 inhibitors. *Oncol Res Treat*. 2025;48:360–71. 40073848 10.1159/000545210

[27] Kawachi H, Fujimoto D, Morimoto T, et al. Early depth of tumor shrinkage and treatment outcomes in non-small cell lung cancer treated using nivolumab. *Invest New Drugs*. 2019;37:1257–65. 30937690 10.1007/s10637-019-00770-y

[28] Hirasawa Y, Kubota Y, Mura E, et al. Maximum efficacy of immune checkpoint inhibitors occurs in esophageal cancer patients with a low neutrophil-to-lymphocyte ratio and good performance status prior to treatment. *Anticancer Res*. 2024;44:3397–407. 39060084 10.21873/anticanres.17160

[29] Chen Q, Zhai B, Li J, et al. Systemic immune-inflammatory index predict short-term outcome in recurrent/metastatic and locally advanced cervical cancer patients treated with PD-1 inhibitor. *Sci Rep*. 2024;14:31528. 39732889 10.1038/s41598-024-82976-6 PMC11682050

[30] Carroll TM, Chadwick JA, Owen RP, et al. Tumor monocyte content predicts immunochemotherapy outcomes in esophageal adenocarcinoma. *Cancer Cell*. 2023;41(1222–41):e7. 10.1016/j.ccell.2023.06.006 PMC11913779 37433281

[31] Hu Y, Xie C, Yang H, et al. Assessment of intratumoral and peritumoral computed tomography radiomics for predicting pathological complete response to neoadjuvant chemoradiation in patients with esophageal squamous cell carcinoma. *JAMA Netw Open*. 2020;3:e2015927. 32910196 10.1001/jamanetworkopen.2020.15927 PMC7489831

[32] Brancato V, Garbino N, Mannelli L, et al. Impact of radiogenomics in esophageal cancer on clinical outcomes: a pilot study. *World J Gastroenterol*. 2021;27:6110–27. 34629823 10.3748/wjg.v27.i36.6110 PMC8476334

[33] Rakaee M, Tafavvoghi M, Ricciuti B, et al. Deep learning model for predicting immunotherapy response in advanced non-small cell lung cancer. *JAMA Oncol*. 2025;11(2):109–18. 10.1001/jamaoncol.2024.5356 PMC11843371 39724105

[34] Ogino S, Nishihara R, VanderWeele TJ, et al. Review article: the role of molecular pathological epidemiology in the study of neoplastic and non-neoplastic diseases in the era of precision medicine. *Epidemiology*. 2016;27:602–11. 26928707 10.1097/EDE.0000000000000471 PMC4892980

[35] Kosumi K, Baba Y, Yamamura K, et al. Intratumour *Fusobacterium* nucleatum and immune response to oesophageal cancer. *Br J Cancer*. 2023;128(6):1155–65. 10.1038/s41416-022-02112-x PMC10006219 36599917

