# Supplementary material for: Early Tumor Shrinkage and Clinical Outcomes for Esophageal Squamous Cell Carcinoma Patients Treated with Immune Checkpoint Inhibitors: Real-World Observational Study
Source: Ann Surg Oncol. 2026 May 27;33(9):8030–40. doi: 10.1245/s10434-026-19849-x (PMC13452839; doi:10.1245/s10434-026-19849-x)
Supplement: Supplementary file 1 [file 10434_2026_19849_MOESM1_ESM.docx]

| Supplementary Table 1. Clinical and pathological characteristics, and early tumor shrinkage among esophageal squamous cell carcinoma cases with ICI regimens | | | | |
| --- | --- | --- | --- | --- |
|  | Early tumor shrinkage | | | |
|  | Univariable OR |  | Multivariable OR |  |
|  | (95% CI) | *P*-value | (95% CI)^a^ | *P*-value |
| Age (for 10-year increment) | 1.29 (0.80-2.12) | 0.30 |  |  |
| Sex |  |  |  |  |
| Female | 1 (referent) | 0.48 |  |  |
| Male | 1.44 (0.52-4.02) |  |  |  |
| Body mass index (for 1-kg/m^2^ increment) | 0.94 (0.83-1.07) | 0.37 |  |  |
| ECOG PS |  |  |  |  |
| 0 | 1 (referent) | 0.097 | 1 (referent) | 0.26 |
| 1-2 | 5.00 (0.56-44.4) |  | 3.36 (0.34-33.2) |  |
| Tumor location |  |  |  |  |
| Ce | 1 (referent) | 0.64 |  |  |
| Te | 0.75 (0.22-2.53) |  |  |  |
| PD-L1 TPS |  |  |  |  |
| 0≤TPS<1 | 1 (referent) | 0.78 |  |  |
| 1≤TPS | 1.20 (0.34-4.20) |  |  |  |
| Indication |  |  |  |  |
| Unresectable | 1 (referent) | 0.24 |  |  |
| Recurrence | 0.62 (0.29-1.37) |  |  |  |
| Treatment line |  |  |  |  |
| 1 | 1 (referent) | 0.18 |  |  |
| 2≤ | 3.92 (0.42-36.3) |  |  |  |
| Albumin (for 1-g/dL increment) | 0.69 (0.32-1.43) | 0.32 |  |  |
| Creatinine (for 1-mg/dL increment) | 1.91 (0.35-10.9) | 0.45 |  |  |
| C-reactive protein (for 1-mg/dL increment) | 1.19 (0.97-1.51) | 0.10 |  |  |
| SCC antigen level (for 1-ng/mL increment) | 0.99 (0.88-1.10) | 0.82 |  |  |
| Neutrophil-to-lymphocyte ratio  (for 1-unit increment) | 1.08 (0.95-1.25) | 0.25 |  |  |
| Lymphocyte-to-monocyte ratio  (for 1-unit increment) | 1.01 (0.95-1.11) | 0.69 |  |  |
| Platelet-to-lymphocyte ratio  (for 1-unit increment) | 1.00 (0.99-1.00) | 0.97 |  |  |
| Systemic immune-inflammation index  (for 1-unit increment) | 1.00 (1.00-1.01) | 0.62 |  |  |
| C-reactive protein to albumin ratio  (for 1-unit increment) | 1.68 (0.93-3.49) | 0.088 | 1.50 (0.76-3.25) | 0.25 |
| Prognostic nutritional index  (for 1-unit increment) | 0.96 (0.90-1.01) | 0.13 |  |  |
| ^a^ Multivariable logistic regression models were used to identify independent risk factors for early tumor shrinkage. The multivariable models included variables showing a univariable association (*P*-value<0.1) with early tumor shrinkage.  Abbreviations: CI, confidence interval; ECOG PS, Eastern cooperative oncology group performance status; ICI, immune checkpoint inhibitor; OR, odds ratio; SCC, squamous cell carcinoma; TPS, tumor proportion score. | | | | |
